# Managing PVR in the Era of Small Gauge Surgery

**DOI:** 10.1155/2021/8959153

**Published:** 2021-12-04

**Authors:** Manish Nagpal, Rakesh Juneja, Sham Talati

**Affiliations:** ^1^Department of Retina and Vitreous, Retina Foundation, Ahmedabad, India; ^2^Department of Retina and Vitreous, Juneja Superspeciality Eye Hospital, Bilaspur, Chhattisgarh, India

## Abstract

Proliferative vitreoretinopathy (PVR) is the leading cause of failed rhegmatogenous retinal detachment (RRD) surgery. Based upon the presence of clinical features and due to associated underlying risk factors, it is classified into various grades based upon its severity and extent of involvement. Despite excellent skills, flawless techniques, and high-end technology applied in the management of RRD, PVR still occurs in 5–10% of cases. Due to the advancements in wide angle viewing systems, advance vitrectomy machines and fluidics, early identification, use of long-term heavy silicon oil tamponades, high-speed cutters, small-gauge vitrectomies, use of perfluorocarbon liquid (PFCL), and small-gauge forceps and scissors, the success rate in the management of PVR has increased leading to improved anatomical outcomes. However, functional outcomes do not correlate well with improved anatomical outcomes. Various complications occur after RRD repair that are responsible for re-retinal detachment and recurrence of PVR. This article highlights causes, risk factors, classification, grading, diagnosis, and approach to management of PVR and post-PVR surgery complications.

## 1. Introduction

**Figure 1:**
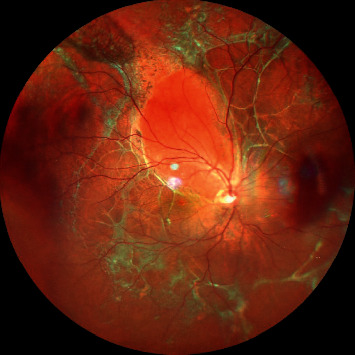
Wide-field color fundus photograph showing retinal detachment with multiple areas of intraretinal and subretinal fibrosis suggestive of proliferative vitreoretinopathy (PVR) changes.

In 1983, on the basis of “massive vitreous traction” or “massive periretinal proliferation,” the Retina Society Terminology Committee put forward a classification and Proliferative Vitreoretinopathy (PVR) was identified as an independent clinical entity [1–4]. It leads to growth and contraction of cellular membranes within the vitreous cavity and on both sides of the retinal surface leading to intraretinal fibrosis and failed rhegmatogenous retinal detachment (RRD) repair surgery (Figure 1) [5, 6]. PVR can manifest in various ways like traction, wrinkling of retinal surfaces, rolled edges, starfolds, and retinal shortening. In the past 2–3 decades, even with the evolution of small-gauge and high cut rate vitrectomies, the overall incidence of PVR still remains the same ranging 5–10% as mentioned in literature by various studies causing 75 percent of all primary surgical failures [6–8]. Henceforth, it is very important to identify the development of PVR, the clinical signs, and subtle risk factors and intervene as early as possible for its management because despite best of the efforts and complex long duration surgery and efforts, majority of eyes suffer complications and low vision [9–11].

## 2. Pathophysiology

PVR develops through a very complex process that involves humoral and cellular factors. The retinal pigment epithelial (RPE) cells, glial cells, fibroblasts, and macrophages act as nidus for its pathogenesis [12]. The various risk factors lead to for membrane formation, ischemia, and subsequent cell death. Cell death triggers a break down in the blood-retinal barrier (BRB) thereby facilitating the influx of chemotactic and mitogenic factors that permit cell proliferation, migration, extracellular matrix deposition, and contraction. Cellular proliferation occurs due to inflammatory mediators and growth factors in vitreous [13–15]. Owing to the production of these pathogenic components, breakdown of BRB, along with retinal tears, and surrounding detachment, there occurs inwards movement of RPE and glial cells causing retinal contraction and other varied features of PVR. Gravity acts as a major factor causing settlement of migrated RPE cells along with other inflammatory mediators that is responsible for increased incidence of PVR in inferior RDs. All these underlying pathogenic mechanisms along with inflammation-induced apoptosis make PVR self-propagatory, complicating retinal detachment surgery leading to blindness even after a successful uneventful retinal attachment surgery [16–20].

### 2.1. Causes, Risk Factors, and Clinical Signs for Diagnosis of PVR

**Figure 2:**
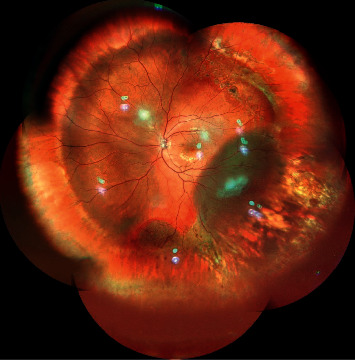
Montage color photograph showing inferotemporal subretinal fluid suggestive of recurrent retinal detachment with attached macula and buckle indentation effect status after buckle surgery.

PVR may be present spontaneously with primary retinal detachment or may develop even after retinal detachment surgery (Figure 2). Various causes and risk factors have been identified that if present can lead to increased incidence of PVR, few weeks to months after primary surgery. These include choroidal detachment, failed RRD surgery, or multiple retinal surgeries, aphakia, vitreous hemorrhage, high vitreous protein levels, positive smoking history, preoperative retinal folds, horseshoe retinal tears exposing three disc diameters or more of RPE, uveitis, giant retinal tear, intra/postoperative hemorrhage, retinectomy, cryopexy, extensive laser, and injection of air [21–27]. These ignite the cascade of events by igniting movement of RPE cells into vitreous cavity leading to complex pathogenic mechanism resulting in formation of membrane that eventually tends to contract causing PVR complication. The presence of these risk factors during pre-, intra-, or postoperative period warrants the need of a close followup to enable early detection of PVR and needful intervention.

**Figure 3:**
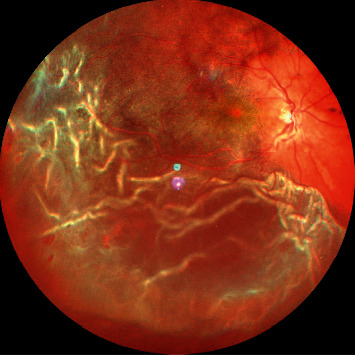
Wide-field color fundus photograph showing retinal detachment with wrinkling of retinal surface (PVR grade B) and multiple retinal breaks seen temporally.

**Figure 4:**
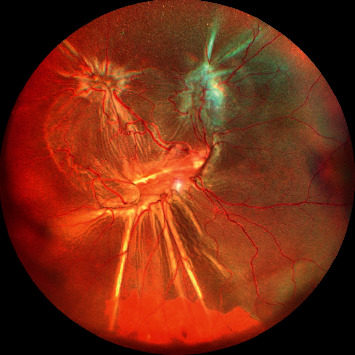
Wide-field color fundus photograph showing total retinal detachment fixed retinal folds suggestive of PVR grade C.

**Figure 5:**
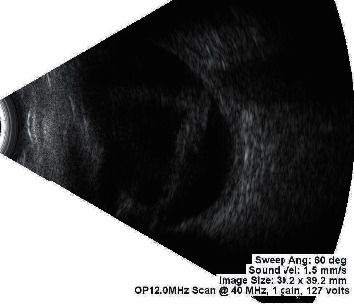
Ultrasonography (B-scan) report suggestive of membranous echoes in vitreous cavity with restricted movements and attachment to optic disc is suggestive of open funnel retinal detachment.

Initially preretinal PVR adopts an immature appearance and consistency. Later on, by 6 to 8 weeks, the membrane becomes more mature, taking on a white, fibrotic appearance. In this stage, PVR can be easily seen clinically and causes retina to become stiff and immobile [28, 29]. These membranes tend to contract over a period of time causing wrinkling, folds, local contraction, and rolled posterior edges (Figure 3). With time, these membranes tend to become more severe causing fixed rigid folds “starfolds” more predominantly in inferior quadrants and tend to bridge with each other further reducing the mobility of retina (Figure 4). Eventually it progresses posteriorly and with PVD leads to formation of advanced PVR causing retina to acquire a funnel shaped appearance (Figure 5).

Various tools in the form of slit lamp biomicroscopy and indirect ophthalmoscopy along with the use of various lenses in eyes with clear media and using B-scan in cases of opaque media help in the diagnosis of PVR along with various risk factors. It helps in meticulous planning of surgical approach. Early intervention is better as PVR often leads to substantial vision loss and a poor visual outcome.

### 2.2. Classification of PVR

In 1983, PVR as a significant clinical entity was noted and a classification for grading of PVR was put forward by the Retina Society Terminology Committee [30–32]. It was initially the most widely used grading system based on clinical signs and geographical distribution pattern. It had numerous limitations. It failed to take into account the anteroposterior epiretinal proliferation and degree of cellular proliferation. In 1989, Silicone Study classification expanded the initial contributions by incorporating (1) membrane location, (2) clinical severity, and (3) membrane geometry [33]. It was difficult to be used in clinical practice and moreover it failed to offer a significant advantage in terms of decision making with regards to treatment. Hence, in 1991, a revised classification was put forward by Machemer et al. [34], which took into consideration more factors while grading the severity of PVR and was widely accepted. It is very essential to classify and grade PVR as it helps in better planning and management of PVR.

### 2.3. Prevention of PVR

The most important aspect to prevent development of PVR is the early identification of various risk factors that are held responsible. The key feature is to pick up early various subtle signs and along with increased awareness that can help surgeon to modify the plan of management thus helping to prevent this serious complication. If early PVR is noted, it is better to proceed with combined scleral buckling and vitrectomy rather than a single procedure of the two, along with the use of long-term heavy silicon oil tamponades.

### 2.4. Diagnosis of PVR

Broadly PVR can be divided into two groups: (1) preexisting with rhegmatogenous retinal detachment (RRD). This is seen usually with long standing RRDs. (2) PVRs that occur after primary surgery for RRDs: this usually occurs after a period of 4–6 weeks of initial surgery. Initially retina seems attached with some visual gain which later tends to deteriorate with the development of PVR. During examination, PVR is identified by retinal traction caused by retinal membranes. In most of the cases of PVR, inferior retina is affected more due to gravity-based deposition of RPE cells. Vitreous haze along with the release of pigment cells in vitreous cavity and over the surface of retina can be seen. Posterior PVR is detected by starfolds with folds radiating from a central area of contracted retina, and more diffuse folds and later on subretinal membranes are also seen beneath the retina. These folds may even take an annular configuration pulling the retina over the optic disc [29]. Membranes may be circumferential at or posterior to the vitreous base. With contraction at the posterior edge of the vitreous base, the anterior retina may be stretched centrally while the posterior retina is thrown into radial folds extending from the vitreous base posteriorly.

### 2.5. Diagnostic Procedures for PVR

**Figure 6:**
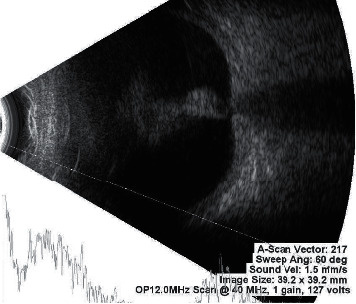
Ultrasonography (B-scan) report suggestive of membranous echoes in vitreous cavity with restricted movements and firm attachment to optic disc (T-pattern) is suggestive of closed funnel retinal detachment.

Diagnosis of the presence of PVR and its grading based on available classification systems is done with the help of indirect ophthalmoscopy examination with +20D lens. A thorough examination of retina is conducted and grading of PVR is done with the aid of retinal drawing. This can be done in presence of clear media allowing clear view of retina. However, in presence of media opacity obscuring view of retina and henceforth, disabling retinal examination, B-scan is the preferred diagnostic tool. Examiner asks patients to move their eyes while performing B-scan to identify the mobility of detached retina. In the absence of PVR, in a case of RRD, the retina has good mobility on B-scan. In a case of RRD with PVR, the flaps of the retina may assume a “V” pattern at the optic disc with very limited retinal mobility as they approach the optic nerve (open funnel retinal detachment) (Figure 5). With more severe PVR, the retina may assume a “T” pattern on ultrasound at the optic disc (closed funnel retinal detachment) with the detached flaps of retina fused together anterior to the disc, only opening more anterior to the disc with the anterior immobile retina completing the top bar of the “T” (Figure 6).

### 2.6. Surgery for PVR

The mainstay for management of PVR is surgical intervention. With the evolution of surgical techniques, better instruments, fluidics, facilities, wide angle viewing systems, small-gauge vitrectomies, and heavier silicon oil tamponades, there is a significant rise in success rate of PVR surgeries. PVR may present with single clinical feature or multiple features in different cases. There may be only a starfold at a single location, fixed membranes leading to funnel shaped retina, immobility of retina, contraction, and retinal stiffening and shortening. The main goal of management is to relieve the traction and to reattach the retina. However, in cases with severe PVR, additional maneuvers are required to relieve traction in order to reattach the retina and prevent redetachment [6, 7, 35]. These goals can be better achieved with the help of a combined and meticulously done scleral buckle and vitrectomy with long-term silicon oil tamponade and also to prevent retinal redetachment and recurrence of PVR to as much extent as possible [36, 37].

Although vitrectomy with removal of entire remnant vitreous, posterior hyaloid, and fibrinous and cellular elements causing traction is the core concept in management of PVR, scleral buckling also has a significant role when treating PVR detachments. Scleral buckles relieve both anteroposterior traction and circumferential traction. In a case of RRD with PVR, encircling bands that support the entire vitreous base are more useful than segmental elements and are frequently used along with PPV [6]. However, Yao et al. in their study have reported to achieve high rates of anatomic success using scleral buckling alone in chronic detachments with PVR [38].

Many surgeons believe and have also published in literature that vitrectomy along with silicon oil is enough for the management of PVR [39]. However, there is enough evidence in literature that suggests that a combined vitrectomy with silicon oil tamponade along with scleral buckling gives better results and higher success rate. Vitrectomy directly allows the surgeon the access to the entire pathological insult going in the removal of, proliferating, and migrating epithelial cells, blood, fibroblast, peeling of membranes causing traction, folds, wrinkling, shortening, and contraction of retina. With the availability and worldwide use of wide angle viewing systems, advanced fluidics, phacoemulsification techniques for the management of lens with IOL implantation, use of direct and indirect contact lenses that enable a very wide crisp panoramic view of retina, small-gauge vitrectors, high-speed cut rates allowing less traction and no incarceration of retina, heavy silicon oil for adequate tamponade, heavy liquids perfluoro-n-octane carbon liquid which is heavier than water (PFCL) causing displacement of subretinal fluid, fiber-optic chandelier illumination allowing access to periphery in great details with bimanual approach to surgeon, small-gauge advance sharp, versatile forceps and scissors allowing smooth bimanual dissection, use of active aspiration by small-gauge soft tip cannulas, and membrane scrappers, the success rate in the management of complex PVRs has gone up to a significant extent.

For PVR surgery, either local or general anesthesia is acceptable. However, owing to prolonged duration of surgery, also based on type and grade of PVR, present and various other patient related factors, and the comfort of the surgeon and the patient, the type of anesthesia is decided as per case.

**Figure 7:**
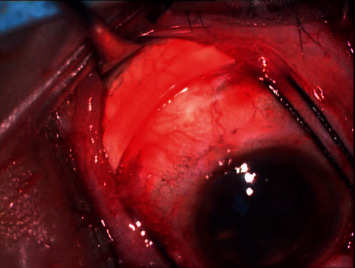
Intraoperative image showing 240 mm encircling silicon band placed underneath conjunctiva which is applied 360 degrees to give support to vitreous base.

**Figure 8:**
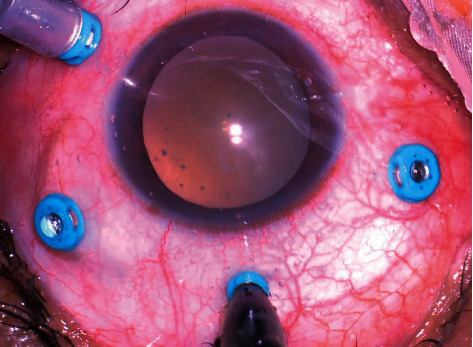
Intraoperative image showing 25 G chandelier illumination placed at 12 o'clock along with other three 25 G cannulas placed in superonasal, superotemporal, and inferotemporal quadrants.

After a thorough meticulous examination, the severity and stage of PVR are judged, and the approach is planned. The approach consisting of combined scleral buckle with vitrectomy gives more effective outcomes. If a scleral buckle is planned, conjunctiva is opened by limbal peritomy and an encircling 360° scleral buckle is put (Figure 7). Usually to provide long-term support, narrower bands are preferred. Once the scleral buckle part is done, 4 port pars plana entry is done by 23- or 25-gauge trocar cannula. Infusion is attached at the inferotemporal quadrant and superonasal and superotemporal ports are used for endoillumination and cutter. The 4th port is placed based on surgeons' choice as per need of the case for chandelier illumination in order to obtain a good panoramic view and allow the surgeon to perform bimanual surgery at ease (Figure 8). Care needs to be taken to avoid pars ciliaris entry and surgeon should ensure proper entry in vitreous cavity and to avoid entry into suprachoroidal or subretinal space.

**Figure 9:**
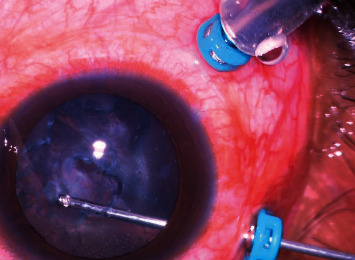
Intraoperative image showing pars plana lensectomy being done with the help of a 25 G vitrectomy cutter.

One should definitely try that natural lens should be preserved. The natural lens always tends to become cataractous with silicon oil and later can be comfortably removed as a separate procedure or during a combined sitting with silicon oil removal. But, if the cataract is significant enough obscuring the view during retina surgery, it needs to be removed along with retina surgery. Removal of lens along with retina surgery also allows good and thorough shaving of vitreous base. Lens can be removed either by phacoemulsification/small incision cataract surgery of pars plana lensectomy with or without IOL implantation based upon the situation (Figure 9). One must do an inferior iridotomy if IOL is not implanted in the same sitting and the patient is left aphakic to avoid silicon oil coming in contact with corneal endothelium [40].

**Figure 10:**
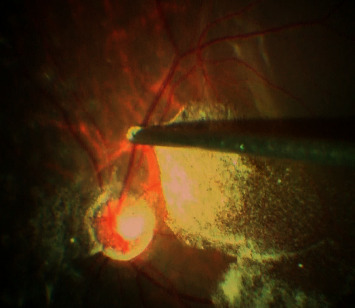
Intraoperative image showing triamcinolone-assisted posterior vitreous detachment (PVD).

**Figure 11:**
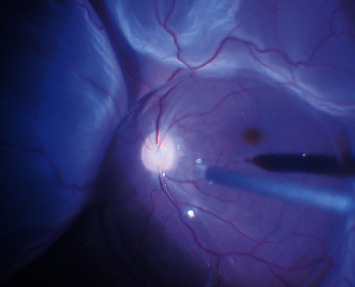
Intraoperative image showing injection of perfluoro carbon liquid (PFCL) stabilizing the posterior pole and pushing the subretinal fluid in the periphery in a case of bullous retinal detachment.

After dealing with lens and obtaining a clear view of retina, meticulous removal of vitreous is done along with the removal of posterior hyaloid. Intravitreal injection of triamcinolone acetonide helps in identification of vitreous to avoid any remnant vitreous and posterior hyaloid (Figure 10). 23-gauge vitrectomy is the system of choice as it allows ease of access to the entire vitreous base with good fluidics and a sutureless postoperative closure. With the aid of high-speed vitrectomy cutters and scleral depressors, base shaving is also done clearly with minimal to no vitreous incarceration after stabilizing the posterior pole with PFCL by displacing subretinal fluid (Figure 11).

**Figure 12:**
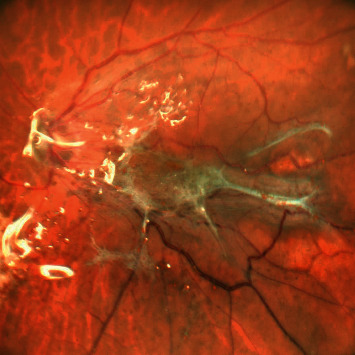
Central color fundus photograph showing Macular Pucker in a case of silicon oil-filled vitrectomized eye which was operated on for retinal detachment with proliferative vitreoretinopathy changes.

Once the vitreous is removed, it is extremely important to remove all folds, wrinkling, and contraction by identifying the membranes that are responsible for causing them which can be facilitated by using brilliant blue dye (BBD). BBD helps to stain the membranes that can be peeled by forceps or trimmed with cutter as per situation. It is very important to identify all membranes and to remove them all, to enable retina to become mobile again (Figure 12). Due care should be taken that PFCL should not go subretinal through open breaks/tears. It can happen if membranes are still persisting preventing complete flattening of retina.

In long-standing PVRs, subretinal bands are noted that prevent retinal flattening even after meticulous removal of all preretinal and retinal surface membranes. This warrants the need of a small retinectomy and removal of these bands assisted with forceps. Once all subretinal bands are removed and even after that retina fails to flatten due to severe contraction or shortening owing to chronicity or long-standing contraction, then retinectomy is needed to achieve retinal flattening. Small-gauge soft tip cannulas are inserted into retinectomies or breaks and subretinal fluid is aspirated by active suction along with fluid-air exchange enabling the surgeon to achieve retinal flattening [41–50].

**Figure 13:**
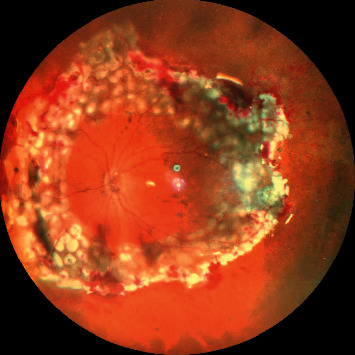
First day postoperative image showing 360-degree lasered retina in a case of extensive PVR.

After retinal flattening, chorioretinal apposition is needed which is done by a good endolaser photocoagulation around all the breaks, retinotomies, and retinectomies followed up at 360° endolaser barrage around the peripheral retina (Figure 13). Excessive laser and heavy burns should be avoided as they may act as precursor of re-PVR and recurrence. Laser is preferred over cryotherapy as cryo leads to excessive inflammation, more complications leading to cellular proliferation, and risk of recurrence of PVR. In certain situations where laser photocoagulation cannot be performed then cryo will serve the purpose of chorioretinal adhesion. Inability to achieve visible laser burns signifies that retinal flattening is still not achieved [50].

Once all breaks are sealed with laser, next step involves the use of a tamponade. Although both gas and silicon oil can be used as tamponading agents, out of both, silicon oil provides long-term and more adequate tamponade in PVRs. Literature suggests that most surgeons prefer silicon oil as tamponade in the management of PVR. The use of gas is associated with restricted air travel and risk of ocular hypertension and an inadequate tamponade leading to more chances of recurrence [51, 52]. Some surgeons prefer direct PFCL-silicon oil exchange but the majority usually prefer fluid-air-silicon oil exchange. Silicon oil is injected with a pressure-assisted delivery system along with the use of a silicon oil tip cannula. Once silicon oil is injected, the infusion pressure is reduced to maintain appropriate intraocular pressure. Once silicon oil touches the sclerotomy port, it pushes the residual air out of the eye and then silicon oil is further injected keeping a close watch on intraocular pressure, as it should be adequate enough.

Various less viscous forms of standard silicon oil are available like 1000, 1300, and 1500 cSt (centistokes). Slightly more viscous form 5000 cSt is also available. Few surgeons prefer less viscous form and few surgeons more viscous form. However, if the breaks are not well closed and residual traction is still present, the oil of any viscosity will seep through and enter into subretinal space through open breaks. The standard silicon oils are lighter than water.

Standard silicon oils fail to provide perfect apposition between oil bubble and peripheral inferior retina causing a gap, which can get filled with proliferating residual cells and debris and leading to recurrence more commonly involving inferior retina. This condition can be avoided by use of heavy fluorinated silicon oil which provides a better tamponade particularly after inferior relaxing retinectomy. However, long-term retention side effects of heavy fluorinated silicon oil are yet known, so they are preferably removed within a time frame of 3 months [53–66]. Once surgery is done, dilute injection of antibiotic is injected in subtenon's space along with anesthetic agent to counter postoperative pain and infection. Conjunctiva is closed if initial peritomy was done for scleral buckle.

**Figure 14:**
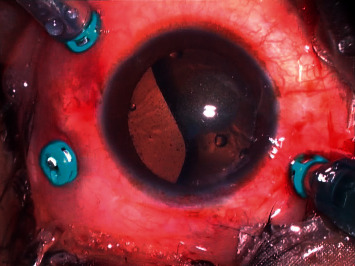
Intraoperative image showing silicon oil removal being done with 25G vitrectomy system.

Usually within an interval of 3 to 6 months based on various factors along with scar maturity, silicon oil removal is done. Long-term retention may lead to certain unwanted complications. However, after silicon oil removal, there is a significant risk of retinal redetachment [67]. Despite a successful surgery with good retinal flattening, many eyes have a poor visual potential and multiple surgeries may be needed if recurrence is noted [68–70]. Cataract almost always occurs with the use of silicon oil and at times oil gets emulsified leading to glaucoma and requires urgent removal. Band keratopathy may occur even if silicon oil is confined in vitreous. Silicon oil removal is carried out as a separate operating procedure in which silicon oil removal is done with a pressure-assisted silicon oil tip cannula by high-pressure active aspiration using advance modern vitrectomy machines (Figure 14). The vitreous compartment is filled with either air or saline as postoperative tamponade [71].

### 2.7. Management after Surgery for PVR

Postoperative prone positioning is advised for approximately 10 hours a day for the first 1 week, which can later be reduced to 4–6 hours/day for the next 3 weeks. The idea behind this is to egress out any residual subretinal fluid by retinal pigment epithelium pump mechanism and to promote chorioretinal adhesion leading to retinal scar formation at the site of laser. Postoperative intraocular pressure rise can be seen which needs immediate intervention by oral and topical antiglaucoma medications to avoid any damage to optic nerve and associated pain. Most common cause of raised intraocular pressure is the overfill of silicon oil and at times if medications fail to bring down pressure to a desired value, then a small volume of oil needs to be aspirated out surgically. The cycloplegics, antibiotics, and anti-inflammatory drugs keep a check on postoperative infection and inflammation.

### 2.8. Complications following Surgery for PVR

Management of PVR is complex and even after a successful retinal attachment complications can occur and patients along with relatives need to be informed along with a written informed consent preoperatively about possible complications. Some intraoperative complications that can occur include bleeding, corneal edema, pupillary constriction, lens clouding, subretinal migration of PFCL and/or oil, gas, choroidal detachment, and choroidal bleeding. Intraoperative bleeding during surgery can be managed by raising the infusion pressure or by applying cautery at the site of bleeding for few minutes to allow clot formation and bleeding to stop. Corneal edema or opacification can develop due to prolonged contact of viscous material and needs epithelial debridement allowing clear visualization during surgery. Pupillary constriction can be managed by use of intraoperative intracameral use of dilating agents or cutting the synechiae or membrane, which is causing obscuration of view. Lens clouding or opacification due to a preexisting significant cataract or due to intraoperative lens touch needs lensectomy or a planned cataract surgery by phacoemulsification or small incision cataract surgery with or without IOL implantation. Subretinal migration of PFCL or oil needs to be approached by making a small meticulous retinotomy and drainage. Choroidal detachment usually happens due to wrong placement of cannula into suprachoroidal space and is managed by securing infusion line through a separate cannula and reinserting misplaced cannula into vitreous cavity.

A close followup is vital as immediate postoperative complications include ocular hypertension, pupillary block glaucoma, shallowing and closure of the anterior chamber, intraocular inflammation, subretinal hemorrhage, and silicon oil in the anterior chamber. Ocular hypertension needs immediate intervention with anti-glaucoma medications. If medical management fails to lower the intraocular pressure, it usually happens due to silicon oil overfill and some amount of oil needs to be extruded to maintain the desired pressure level. Other complications also may need immediate surgical intervention if causing pain and raised intraocular pressure as they may vision threatening and may lead to permanent vision loss.

**Figure 15:**
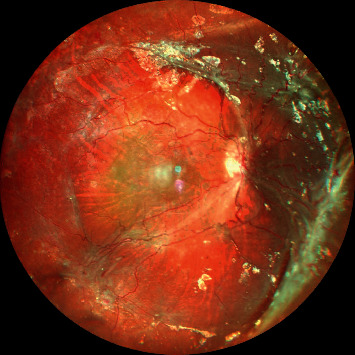
Widefield color fundus photograph showing reretinal detachment under silicon oil in a case of pars plana vitrectomy done for retinal detachment with PVR.

Late complications of PVR surgery include regrowth of membranes causing traction, opening of old breaks, formation of new breaks, recurrent retinal detachment (Figure 15), glaucoma due to emulsified silicon oil, corneal endothelium damage due to silicon oil in anterior chamber leading to band keratopathy, cataract due to silicon oil touch, hypotony-phthisis, squint, double vision due to scleral buckle, infection of scleral buckle, and macular pucker (Figure 12) [72, 73]. For regrowth of membranes and macular pucker, resurgery needs to be planned. Staining is vital to identify any new membranes of remnant ones causing traction and PVR clinical features. Once identified, these membranes need to be peeled to allow retina settle again. Peeling of internal limiting membrane should be done after staining to avoid recurrence of macular pucker. Any new breaks or old opened breaks should be managed by adequate endolaser photocoagulation after settling the retina. Removal of silicon oil is important especially if it is migrating to anterior chamber or causing endothelial toxicity by silicon oil removal surgery. Band-keratopathy needs scraping of cornea along with the application of chelating agent EDTA. In case of a scleral buckle infection or extrusion, surgeon must plan to immediately remove scleral buckle under the cover of antibiotics to prevent further spread of infection. Glaucoma is managed with topical antiglaucoma medications or surgery, and for others reretinal surgery is usually needed and more often leading to very poor visual outcome. All these complications are visually fatal and need to be addressed immediately.

### 2.9. Results of PVR Surgery

Over the past few decades with advancement in techniques, instrumentation, and machines, the success ratio in anatomical management of PVR has increased a lot. Still, many eyes undergo redetachment that requires resurgery. Once macula is detached for more than few days, it is unlikely to recover more than 10–20% of central vision. Henceforth, anatomical success in terms of successful retinal attachment for a period of 6 months cannot be compared as equivalent to functional success [20]. Prolonged duration of PVR or a more severe PVR indicates that visual potential is lost. Despite the best efforts, visual prognosis remains very poor.

## 3. Conclusion

PVR is the leading cause for failure of RRD repair and is identified by the growth and contraction of cellular membranes within the vitreous cavity and on both sides of the retinal surface leading to retinal contraction and fixed starfold formation. Various risk factors have been identified that lead to PVR formation. Despite the best of machines, advances in techniques, instrumentation, and the increased success rate in terms of anatomical reattachment, the real meaningful long-term stable successful visual outcome is yet not achieved and many eyes in long course tend to suffer severe vision loss. Medical therapy is also tried but to date no real success. However, we may hope that, with the continual advancements in techniques and technology going on, some way of restoring long-term meaningful visual potential may soon arrive in the medical world.

## Data Availability

No data were used to support this study.
